# Molecular Signatures of Human Regulatory T Cells in Colorectal Cancer and Polyps

**DOI:** 10.3389/fimmu.2017.00620

**Published:** 2017-05-30

**Authors:** Nor Adzimah Johdi, Kamel Ait-Tahar, Ismail Sagap, Rahman Jamal

**Affiliations:** ^1^UKM Medical Molecular Biology Institute, Universiti Kebangsaan Malaysia, Cheras, Kuala Lumpur, Malaysia; ^2^Faculty of Medicine, Department of Surgery, Universiti Kebangsaan Malaysia, Cheras, Kuala Lumpur, Malaysia

**Keywords:** regulatory T cells, colorectal cancer, gene expression, immune suppression, interleukin

## Abstract

Regulatory T cells (Tregs), a subset of CD4^+^ or CD8^+^ T cells, play a pivotal role in regulating immune homeostasis. An increase in Tregs was reported in many tumors to be associated with immune suppression and evasion in cancer patients. Despite the importance of Tregs, the molecular signatures that contributed to their pathophysiological relevance remain poorly understood and controversial. In this study, we explored the gene expression profiles in Tregs derived from patients with colorectal cancer [colorectal carcinoma (CRC), *n* = 15], colorectal polyps (P, *n* = 15), and in healthy volunteers (N, *n* = 15). Tregs were analyzed using CD4^+^CD25^+^CD127^low^FoxP3^+^ antibody markers. Gene expression profiling analysis leads to the identification of 61 and 66 immune-related genes in Tregs derived from CRC and P patients, respectively, but not in N-derived Treg samples. Of these, 30 genes were differentially expressed both in CRC- and P-derived Tregs when compared to N-derived Tregs. Most of the identified genes were involved in cytokine/chemokine mediators of inflammation, chemokine receptor, lymphocyte activation, and T cell receptor (TCR) signaling pathways. This study highlights some of the molecular signatures that may affect Tregs’ expansion and possible suppression of function in cancer development. Our findings may provide a better understanding of the immunomodulatory nature of Tregs and could, therefore, open up new avenues in immunotherapy.

## Introduction

Regulatory T cells (Tregs) are suppressor cells that play a pivotal role in regulating immune hemostasis and immunological tolerance to self (1). Tregs are present in low numbers (1–2% of lymphocytes) within both CD4^+^ and CD8^+^ populations (2, 3). Their main function is to prevent inappropriate immune responses by suppressing immune effector cells. This is useful for maintaining immune hemostasis in autoreactivity, severe inflammation, and transplantation in patients. However, excessive Tregs’ oversuppression in cancers and tumor environment may lead to undesirable immune tolerance and evasion. The frequencies of Tregs in peripheral blood, lymphoid tissues, and tumor microenvironment have been investigated in many different types of cancers including liver, breast, renal, leukemia, and gastric cancers and were associated with cancer progression and poor prognosis suggesting their critical role in tumor development (4–8). However, contradictory reports on their role exist in the colon, gastric, and head and neck cancers (9–11). The discrepancies could be due to unstandardized antibody markers used in different laboratories and varying terminologies used to describe different subpopulations of Tregs (12). This complicates direct comparison between studies. Despite the availability of many markers associated with Tregs, the most prominent Tregs are CD4^+^CD25^+^CD127^low^FoxP3^+^ (13–15). At the functional level, cytokines produced by Tregs such as elevated levels of IL-10 (16, 17) and TGF-β (18) are also widely used. Various mechanisms contribute to the elevated numbers and suppressive function of Tregs in cancer. However, there are a limited number of studies describing the molecular signatures that may contribute to the underlying Tregs-mediated tolerance and suppression in cancer development.

Colorectal carcinoma (CRC) is the third most common cancer worldwide with an estimated number of 1.4 million (9.7%) cases in 2012 (19). In Malaysia, 2,246 CRC cases (12.3%) were reported in 2011 (20). It is widely recognized that genetic factors play important roles in the pathogenesis of CRC. However, evading immune surveillance is recognized as an emerging hallmark in cancer progression (21). There is considerable evidence to suggest that the immune system plays a protective role in tumorigenesis (22–24). Correlation between serrated polyps and colorectal neoplasia has been largely reported (25–27). Approximately 15–20% of all sporadic CRCs arise *via* the serrated pathway, in which serrated polyps may be the precursor lesions (28, 29). Serrated polyps are classified pathologically according to the World Health Organization criteria as hyperplastic polyps (HPs), sessile serrated adenoma/polyps with or without cytological dysplasia, and traditional serrated adenomas (30). In the context of CRC progression, Tregs’ frequencies and function may be important because high frequencies of Tregs might favor CRC and polyps’ growth or development and influence the course of the disease through enhancing suppression of antitumor immunity. Thus, it is interesting to investigate the molecular signatures of Tregs in CRC and polyps and possible correlation with Tregs influences the disease development.

In this study, we explored the gene expression profiles in CD4^+^CD25^+^CD127^low^FoxP3^+^ Tregs derived from CRC, P, and N samples in order to investigate the molecular signatures that may influence cancer development. We identified a number of differentially expressed Treg transcripts derived from CRC and P patients. We suggest these genes to be relevant for Tregs’ general function. Other transcripts were identified to differ among these groups and might give rise to their phenotypic differentiation. These could potentially be used as biomarkers to discriminate Tregs derived from CRC and P patients.

## Materials and Methods

### Patients and Healthy Volunteers

Ethics approval was obtained from the UKM Research Ethics Committee (reference number UKM 1.5.3.5/244/FRGS/2/2013/SKK01/UKM/03/3). Ten to twelve milliliters of peripheral blood were collected in BD Vacutainer^®^ Heparin Tubes (Becton Dickinson) from 15 healthy volunteers (N), 15 patients with colorectal polyps (P), and 15 CRC patients who were diagnosed in UKM Medical Center, Kuala Lumpur (UKMMC) from 2014 to 2015. N samples were used as a control and classified as the participants who went through endoscopy as part of their annual health screening and were diagnosed as normal. P samples were those with primary polyps including serrated adenoma, adenoma polyps, and dysplasia. The HPs were not included in the samples. For the CRC cases, the samples were collected from the Dukes’ B and Dukes’ C stage. Both groups of patients with CRC and P were histologically confirmed, primary diagnosed, and neither received any form of treatments prior to blood sample collection. The histological stage of the tumor was determined according to the Duke’s staging system. Data including patient clinical history, age, gender, colorectal polyp’s classification, and tumor staging are summarized in Table 1. None of the donors suffered from allergies, autoimmune diseases, and were free from acute or chronic infections. Patients who underwent neoadjuvant treatment or resection were excluded from the study.

**Table 1:** **Clinical data of the patients enrolled in the study**.

Variables	N (*n* = 15)	P (*n* = 15)	CRC (*n* = 15)
Age (range)	55–77	56–80	49–81
Median	62 ± 12	68 ± 10	72 ± 11
**Sex**			
Male	9	9	10
Female	6	6	5
**Race**			
Malay	8	5	9
Chinese	7	10	6
**Classification**			
Serrated adenoma		2	
Tubular adenoma		13	
Dukes’ B			8
Dukes’ C			7

### Lymphocyte Isolation

Peripheral blood mononuclear cells (PBMCs) were isolated by the Ficoll/Paque™ PLUS density gradient centrifugation method (GE Healthcare Life Sciences) as recommended by the manufacturer. PBMCs were counted and frozen instantly in liquid nitrogen until analyzed.

### Antibodies and FACS Analysis

All the antibodies used in this study were purchased from BD Biosciences unless stated otherwise. Tregs were stained using anti-CD4 (-PerCP-Cy 5.5, clone SK3), anti-CD25 (-PE, clone 2A3), anti-CD127 (-Alexa 647, clone HIL-7R-M21), and anti-FoxP3 (-Alexa 488, clone 259D/C7) as recommended in the manufacturer’s protocol. A total of 1 × 10^6^ cells were incubated with 10–20 μl of the fluorochrome-labeled antibodies in the dark at room temperature for 30 min, washed twice, and analyzed on the flow cytometer. The stained PBMCs were loaded onto BD FACSAria™ II system (BD Biosciences) and analyzed to confirm the presence of CD4^+^CD25^+^CD127^low^FoxP3^+^ (Tregs) and CD4^+^CD25^−^ (T responder cells). Cells were analyzed and sorted based on their phenotype to a purity of >90%. Data were analyzed using BD FACSDiva™ Software (BD Biosciences). Sorted cells were collected in 12 mm × 75 mm round-bottom tubes coated with human AB serum (Thermo Fisher Scientific) prior to the addition of Dulbecco’s phosphate-buffered saline (Thermo Fisher Scientific) + 1% Human AB serum (Thermo Fisher Scientific, postsort analysis). Sorted cell populations were centrifuged (100 × *g*, 5 min), the supernatant was carefully removed, and the resulting cell suspensions were divided into three fractions. Fraction 1 was used to determine purity, fraction 2 for FoxP3 staining, and fraction 3 for RNA extraction. Purity was calculated as the number of events in the original sort gate after the exclusion of cell debris. Fraction 2 of the sorted cells was stained for intracellular FoxP3 expression using anti-hFoxP3 (-Alexa 488). This was done on cells that were fixed and permeabilized using FoxP3 staining buffer set (BD Pharmingen™), according to the manufacturer’s instructions. This was a postsort analysis to confirm that Treg populations express FoxP3^+^ phenotype.

### RNA Extraction, Quantification, and Microarray Analysis

Total RNA was extracted from 2 × 10^5^ Tregs using RNeasy micro kit (Qiagen). Quality and integrity of the total RNA were quantified on Agilent 2100 Bioanalyzer (Agilent Technologies). Only those RNA samples with RIN number of ≥7 and of good integrity were used in the microarray analysis. Total RNA (100 ng) was then reverse transcribed and the single-stranded cDNA (sscDNA) was amplified using the WT-Amplification Kit Module (Affymetrix). The purified sscDNA (5.5–15.0 μg) was subsequently fragmented and labeled using the GeneChip^®^ WT Terminal Labeling Kit (Affymetrix). Labeled cDNA (3.5 μg) was then hybridized to the GeneChip^®^ Human Gene 2.0 ST array (Affymetrix) using the GeneChip^®^ Hybridization Oven 640 (Affymetrix) at 60 rotations per minute at 45°C for 16 h. After hybridization, the arrays were washed and stained according to the manufacturer’s protocol using a GeneChip^®^ Fluidics Station 450 (Affymetrix). The arrays were scanned using the GeneChip^®^ Scanner 3000 (Affymetrix).

### Real-time Quantitative Reverse Transcription PCR (RT-qPCR)

Validation of differentially regulated target genes identified by expression array was performed using RT-qPCR. The samples were the same CRC, P, and N samples used for transcriptomic profiling. Random primed cDNA was prepared from total RNA using a high capacity RNA to cDNA kit (Thermo Fisher Scientific). Relative gene expression analysis was carried out using SYBR Green on a 7500 Fast Real-Time PCR system (Thermo Fisher Scientific). Gene-specific primer pairs for qPCR were selected from Primer Bank 25 and listed in Table 2. GAPDH gene was used as an internal control. Results were analyzed using a 7500 Fast Real-Time PCR system software (Thermo Fisher Scientific). Relative mRNA levels were normalized to GAPDH as an internal control gene.

**Table 2:** **List of primers for real-time quantitative reverse transcription PCR**.

Gene	Forward primer	Reverse primer
*CCR4*	AGAAGGCATCAAGGCATTTGG	ACACATCAGTCATGGACCTGAG
*CXCL10*	GTGGCATTCAAGGAGTACCTC	TGATGGCCTTCGATTCTGGATT
*CCR1*	CCTGCTGACGATTGACAGGTA	TCTCGTAGGCTTTCGTGAGGA
*CCR2*	TACGGTGCTCCCTGTCATAAA	TAAGATGAGGACGACCAGCAT
*CCR7*	AAGCGATGCGATGCTCTCTC	TTGCGCTCAAAGTTGCGTG
*CCL1*	CTCATTTGCGGAGCAAGAGAT	GCCTCTGAACCCATCCAACTG
*TRAJ1*	GAGGAGGAGAAACCTAAGGGATT	CCGAGGCTTTAGTGAGCATC
*TRGJP2*	GTCATGAGGATCAGAAGGTTGA	CCAGGCGAAGTTACTATGAGC
*IL10RA*	GAGATCCACAATGGCTTCATCC	TTCTCCAGAGGTTAGGAGGCT
*GAPDH*	TGCACCACCAACTGCTTAGC	GGAAGGCCATGCCAGTGA

### Statistical Analysis

Microarray data analyses were carried out using Expression and Transcriptome Analysis Console (Affymetrix). Data normalization based on median signal intensities was qualified and quantified using Robust Multichip Analysis algorithm, which includes background adjustment, quantile normalization, and summarization. A log_2_ fold change >2 was considered as upregulated genes and a log_2_ fold change <−2 as downregulated genes. Statistical significance was calculated by one-way ANOVA and false discovery rate (FDR) correction (adjust *p*-value based on Benjamin–Hochberg Step-Up FDR-controlling Procedure). A *p* < 0.01 was considered statistically significant.

## Results

### Phenotypic Analysis of Tregs

**Figure 1:**
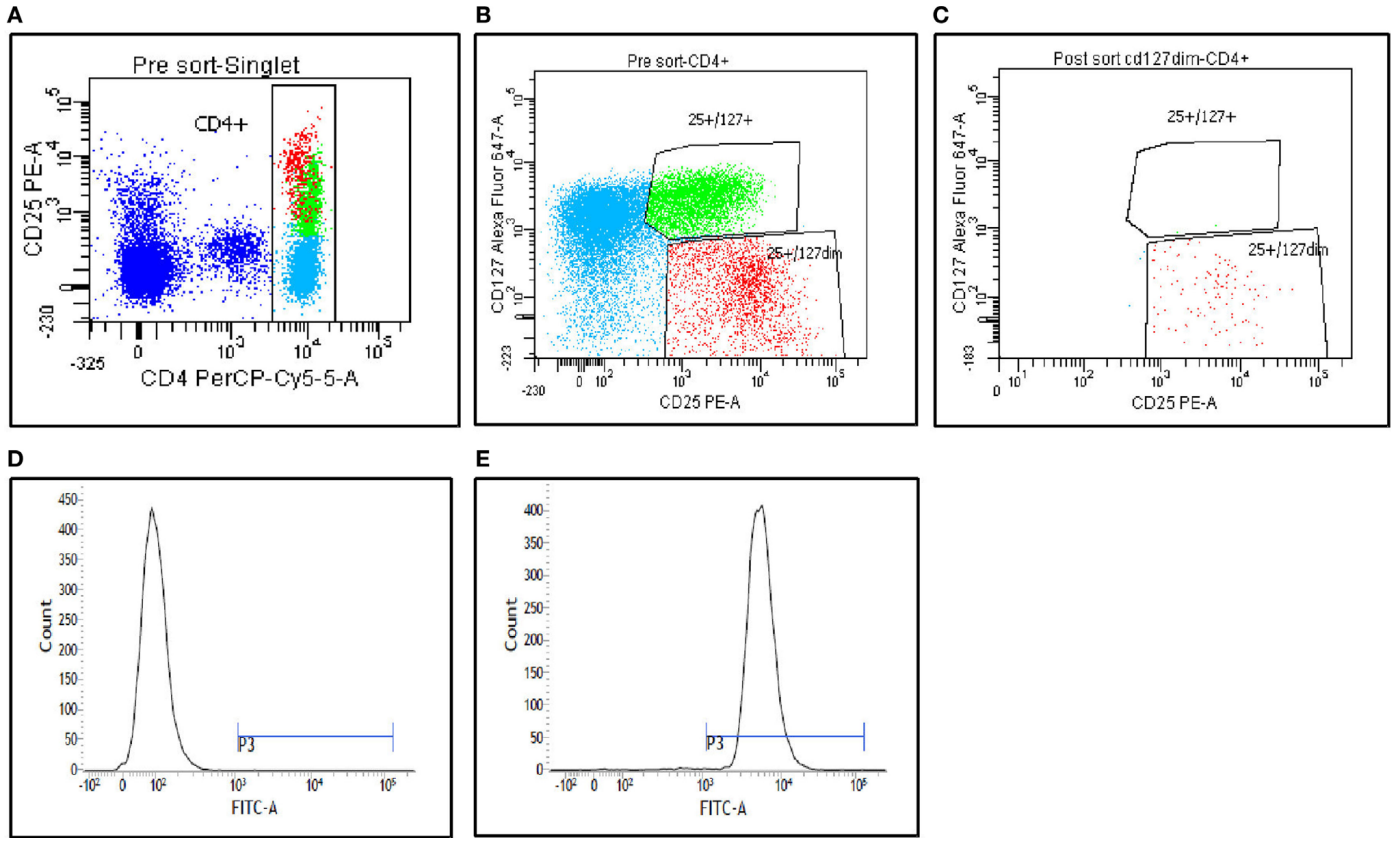
**Peripheral blood mononuclear cells from the patients were stained with CD4, CD25, and CD127, and then analyzed on FACS Aria II (BD Biosciences)**. **(A,B)** Gating strategies used to sort CD4^+^CD25^+^CD127^low^ Tregs and CD4^+^CD25^+^CD127^high^ (non-Tregs). **(C)** Postsort purity determination from fraction 1. **(D,E)** FoxP3 staining analysis of the postsort CD4^+^CD25^+^CD127^low^ Tregs. **(D)** Fluorescence minus one staining. **(E)** FoxP3 staining on postsorted cells.

Figures 1A–C show the gating strategy of CD4^+^CD25^+^CD127^low^ Tregs relative to the CD4^+^CD25^+^CD127^high^ (non-Tregs). The cells were analyzed and sorted from PBMC populations as outlined in the Materials and Methods section. Purity analysis of the fraction 1 postsort CD4^+^CD25^+^CD127^low^ Tregs was performed, and representative data are presented in Figure 1C. The data confirm high purity of more than 90%. The postsorted CD4^+^CD25^+^CD127^low^ Treg populations were also stained for FoxP3 expression. Figures 1D,E show a strong shift indicating a strong Foxp3 expression in these populations. These results support that the isolated T cells were mostly CD4^+^CD25^+^CD127^low^Foxp^+^ Treg populations.

### Analysis of Treg Population

**Figure 2:**
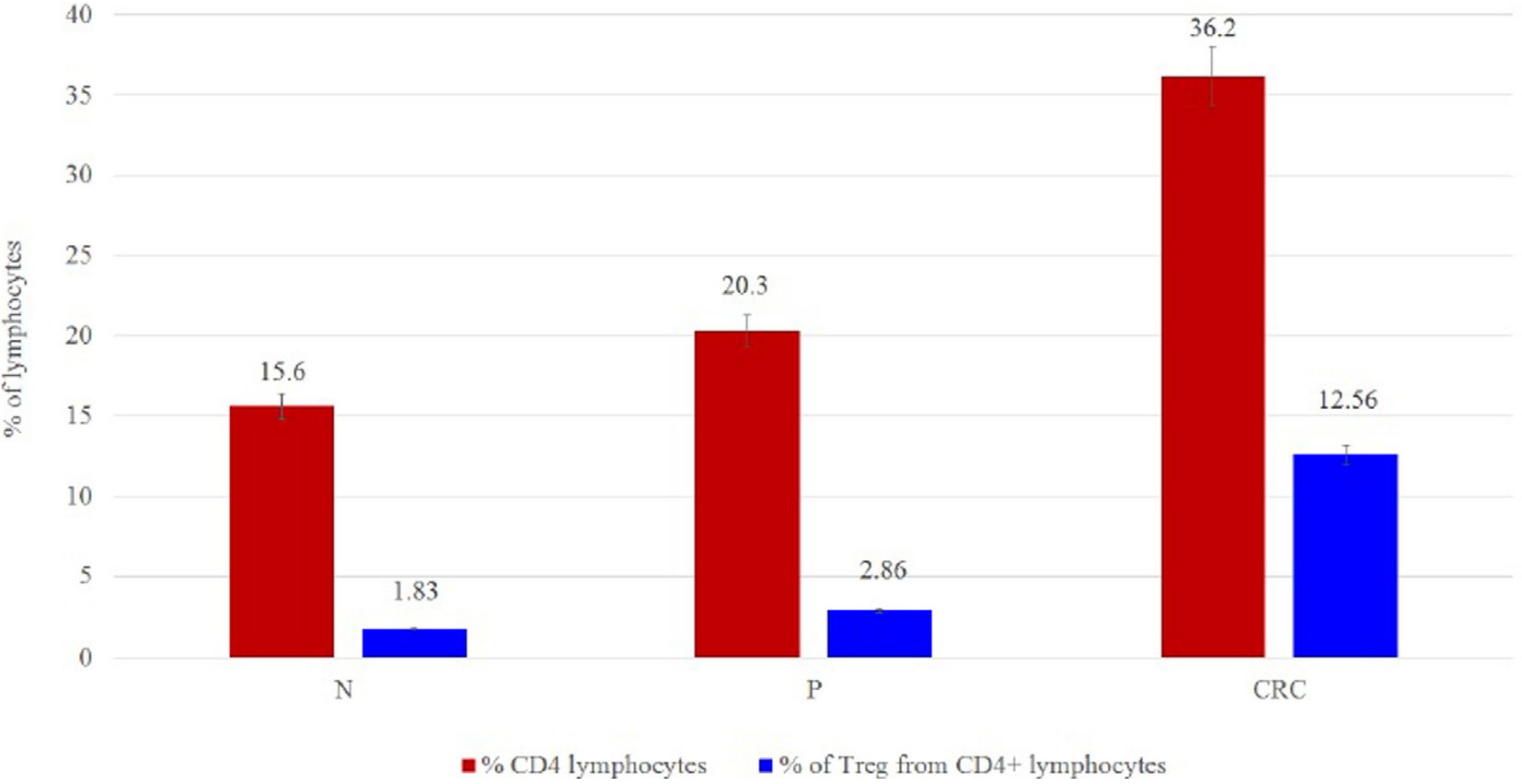
**(A)** Frequency cell counts and percentage of Tregs per 100,000 lymphocytes for N (*n* = 15), P (*n* = 15), and CRC (*n* = 15) samples. The values are given as mean value ± SD, *p* ≤ 0.05. **(B)** Percentage of Tregs from lymphocytes.

Regulatory T cell populations were analyzed and sorted after PBMC treatment as outlined in the Materials and Methods section. The purity of the isolated Tregs was more than 90% and expressed high levels of FoxP3 protein. A significant increase in Treg frequencies was observed in CRC-derived Tregs (12.56%) when compared to P- and N-derived Tregs (2.86 and 1.83%, respectively) (Figure 2).

### Differentially Expressed Genes (DEGs) in CD4^+^CD25^+^CD127^low^FoxP3^+^ Tregs

**Figure 3:**
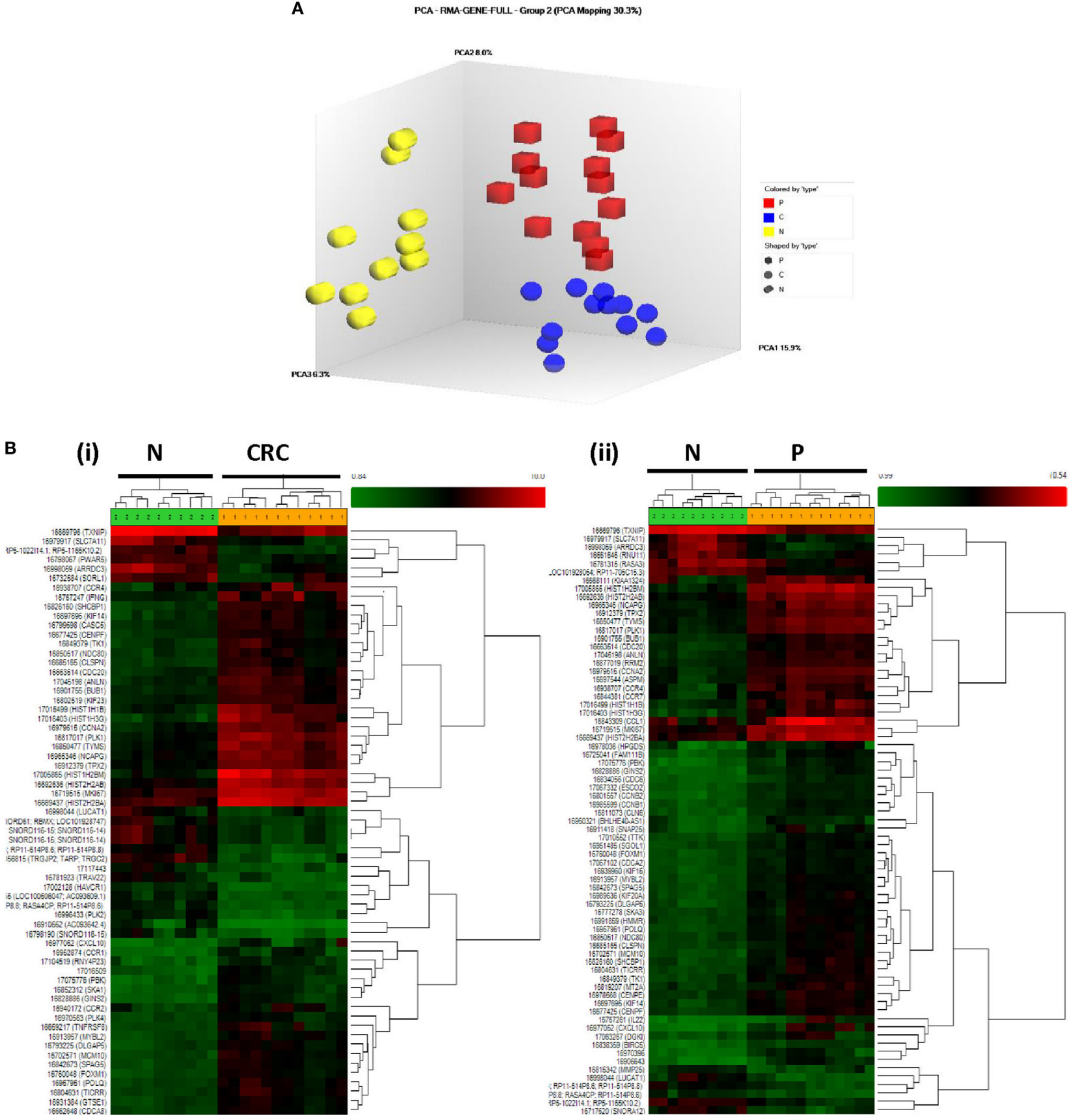
**(A)** Principal component analysis of CRC (blue), P (red), and N (yellow)-derived Tregs. The three Treg populations were clearly segregated. **(B)** Hierarchical clustering of (i) CRC- vs. N-derived Tregs and (ii) P- vs. N-derived Tregs. The one-way ANOVA analysis with false discovery rate correction (*p* < 0.01), log_2_ fold change (>2 or <−2) were considered as upregulated and downregulated genes, respectively.

In order to clarify the possible role played by Tregs in human CRC development, we performed transcriptional profiling of Tregs to identify candidate genes whose expression levels may differ between CRC, P, and N samples. Principal component analysis and hierarchical clustering clearly segregated the Treg transcriptome profiles of CRC and P from N samples, suggesting the three populations to be transcriptionally distinct (Figure 3).

**Figure 4:**
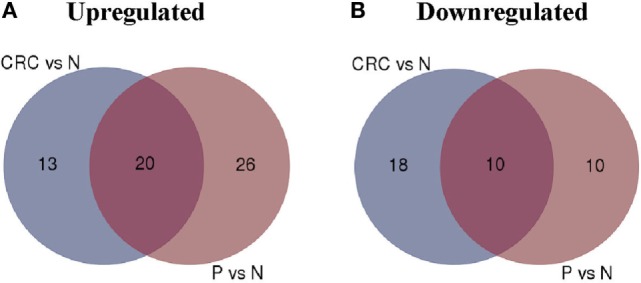
**Venn diagram of colorectal carcinoma (CRC) vs. N and P vs. N. (A)** Number of upregulated genes between CRC- and P-derived regulatory T cells (Tregs). **(B)** Number of downregulated genes in CRC- and P-derived Tregs. Healthy control (N) was used as the baseline.

Analysis of the transcriptome using one-way ANOVA, FDR correction (*p* < 0.01), and absolute log_2_ fold change (>2 or <−2) revealed 689 DEGs (330 upregulated; 339 downregulated) in the CRC-derived Tregs compared with N-derived Tregs (Data Sheet S1 in Supplementary Material). In the P-derived Tregs, 583 genes were differentially expressed (382 upregulated; 201 downregulated) compared to N-derived Tregs (Data Sheet S2 in Supplementary Material). These DEGs were then grouped using gene ontology biological processes, which identified immune-related genes as the most significant DEG function group in CRC- and P-derived Tregs (Data Sheets S3 and S4 in Supplementary Material). We then used the Venn diagram to further delineate the DEGs in both groups. N-derived Tregs were used as the baseline (Figure 4).

The overlapped regions show 20 upregulated and 10 downregulated genes in both CRC- and P-derived Tregs. These genes were differentially expressed in both CRC- and P-derived Treg populations (Table 3). In order to distinguish the two populations, we focused our analysis on the DEGs that appear exclusively in either CRC- or P-derived Tregs using the N-derived Tregs group as the baseline.

**Table 3:** **Top 10 gene list of DEGs that were overlapped in CRC- and P-derived Tregs based on Venn diagram analysis**.

Fold change	*p*-Value	Gene symbol	Description
4.36	0.00038	CCR4	Chemokine (C–C motif) receptor 4
4.32	0.000292	CXCL10	Chemokine (C–X–C motif) ligand 10
4.05	0.000017	IFNG	Interferon, gamma
4.02	6.99E−09	KIF23	Kinesin family member 23
3.91	1.13E−09	CDC6	Cell division cycle 6
−3.51	2.20E−07	TRAJ20	T cell receptor alpha joining 20
−3.51	0.000135	AREG	Amphiregulin
−3.56	2.60E−07	YME1L1	YME1-like 1 ATPase
−3.69	1.32E−08	TRAJ1	T cell receptor alpha joining 1
−5.22	4.36E−09	TXNIP	Thioredoxin interacting protein

In CRC-derived Tregs, the upregulated genes are those of chemokines that are mostly involved in chemotaxis (CCR1, CCR2) and some interleukins that are important in cytokine-mediated signaling pathway in Treg activation (e.g., IL10, SOCS3). There was consistent downregulation of TCRs in CRC-derived Treg samples (Table 4).

**Table 4:** **Top 10 gene list of DEGs that appeared only in CRC-derived Tregs based on Venn diagram analysis**.

Fold change	*p*-Value	Gene symbol	Description
5.16	0.000005	CCR1	Chemokine (C–C motif) receptor 1
4.21	0.000135	CCR2	Chemokine (C–C motif) receptor 2
3.39	0.000004	IFI30	Interferon, gamma-inducible protein 30
3.38	0.000003	IL10	Interleukin 10
2.66	0.000272	SOCS3	Suppressor of cytokine signaling 3
−3.49	1.01E−08	TRAJ4	T cell receptor alpha joining 4
−3.51	2.20E−07	TRAJ20	T cell receptor alpha joining 20
−3.66	6.08E−07	TRAJ3	T cell receptor alpha joining 3
−8.51	0.000013	TRAV22	T cell receptor alpha variable 22
−11.71	1.08E−08	TRGJP2	T cell receptor gamma joining P2

In P-derived Tregs, the DEGs were involved in cell proliferation (e.g., CCNB2 and IL22), binding protein (ANLN), and chemotaxis (CCR7 and CCL1). Significant reduction in expression (*p* < 0.01) was observed in cell surface receptors (TRAJ 14, IL10RA, and TNFR) (Table 5).

**Table 5:** **Top 10 gene list DEGs that appeared only in P-derived Tregs based on Venn diagram analysis**.

Fold change	*p*-Value	Gene symbol	Description
7.32	0.000031	CCR7	Chemokine (C–C motif) receptor 7
6.76	0.000208	CCL1	Chemokine (C–C motif) ligand 1
4.41	2.15E−11	CCNB2	Cyclin B2
4.32	8.00E−11	ANLN	Anillin, actin binding protein
4.11	0.000503	IL22	Interleukin 22
−2.07	0.000087	GAB2	GRB2-associated binding protein 2
−2.31	0.00002	TRAJ14	T cell receptor alpha joining 14
−2.33	0.000005	TNFRSF11A	Tumor necrosis factor receptor superfamily
−2.63	5.75E−07	IL10RA	Interleukin 10 receptor, alpha
−3.65	0.000108	HIST1H2BG	Histone cluster 1

### RT-qPCR Analysis of mRNA Expression

**Figure 5:**
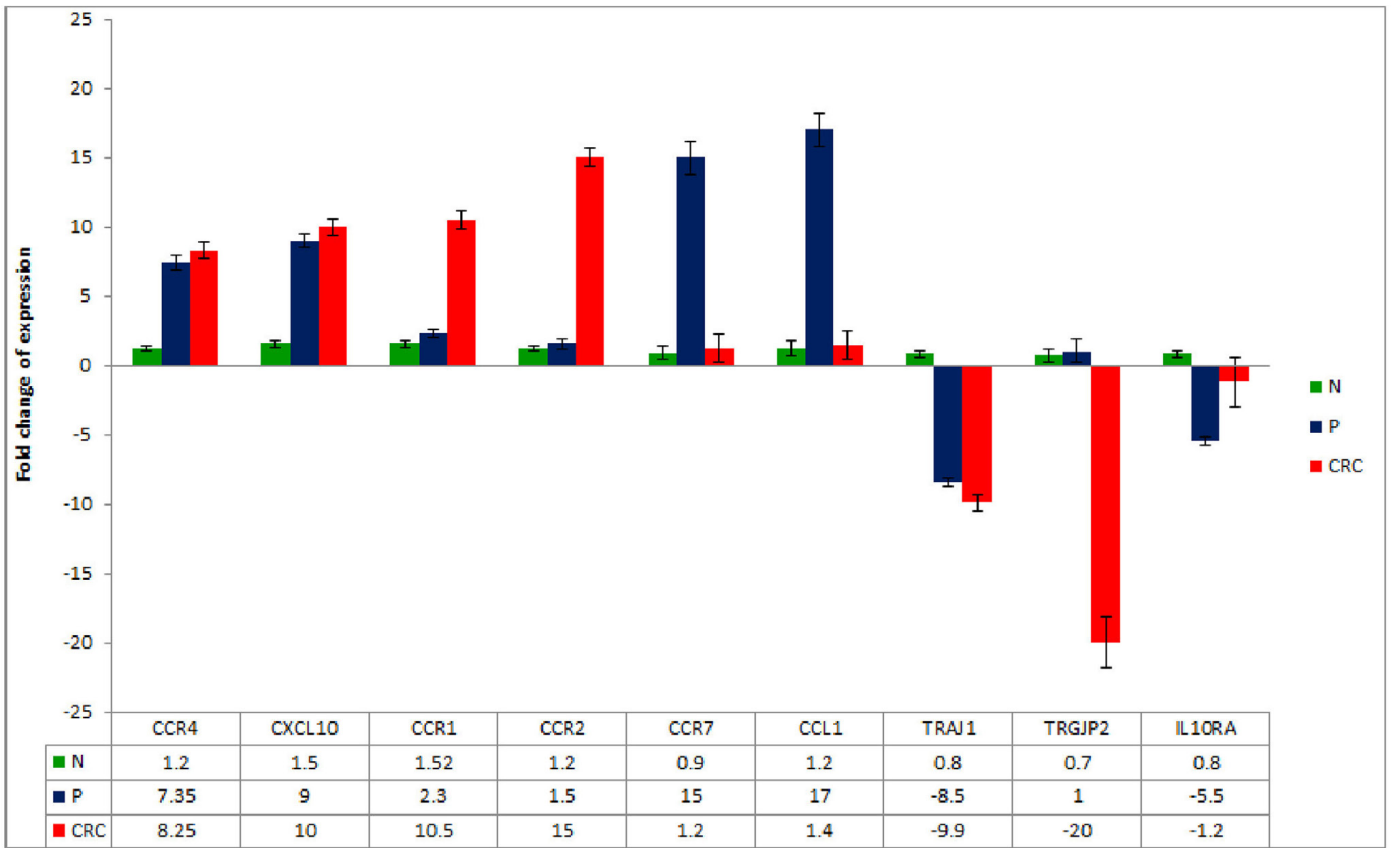
**Real-time quantitative reverse transcription PCR analysis of mRNA expression in CRC-, P-, and N-derived Tregs**. Data are mean ± SD, *n* = 10 for each of the samples.

To further validate the gene expression profiles of the Treg populations, a panel of DEGs was chosen from those highlighted in Tables 3–5 and assessed by RT-qPCR. The analysis confirmed a significant increase in the expression of CCR4, CXCL10, CCR1, CCR2, CCR7, and CCL1 genes and a significant decrease in the expression of TRAJ1, TRGJP2, and IL10RA in the CRC- and/or P-derived Treg populations compared to N-derived Tregs (Figure 5).

## Discussion

CD4^+^CD25^+^CD127^low^FoxP3^+^ Tregs’ frequencies from CRC are higher than P and N samples. This appears to be Tregs’ characteristic feature in most tumors and compares favorably with previously published data (31, 32).

We examined the transcriptome profiles of Tregs derived from CRC, P, and N samples to uncover specific molecular markers and pathways that might be associated with each group. We found that the DEGs which appeared in both CRC- and P-derived Tregs could represent common genes required for Tregs’ suppression of function. It could be that under certain circumstances, some of these genes may trigger immune dysregulation and may also lead to development to polyps or cancer. However, we do not have any clinical data to support this hypothesis.

The common genes identified here include those controlling inflammatory cytokine/chemokine and their receptors. Thus, we observed an upregulation of CCR4 gene expression (both in CRC- and in P-derived Tregs). These findings are consistent with previous studies (33, 34). CCR4 is important in mediating Tregs’ trafficking to different sites of inflammation. Furthermore, CCR4 is specifically expressed by a subset of most suppressive Tregs but not naive Tregs (35). It could be that the CRC- or P-derived Tregs release the CCR4 ligand and thus are able to attract the Tregs’ expressing high levels of CCR4 to the sites of inflammation. Our findings are consistent with previously published data (33, 36) that support the important role of CCR4 in mediating Tregs’ trafficking to different sites of inflammation.

We also observed an upregulation of CXCL10 gene that encodes for IFN-γ-induced protein 10. CXCL10 binds to CXCR3 receptor to promote tumor growth, migration, and invasion of cancer cells in several tumor types (37). CXCR3 is abundantly expressed on other activated effector cells including cytotoxic T-lymphocytes, natural killer cells, and T helper cells. It is possible that the upregulation of CXCL10 in Tregs may affect their homing and migration capacities. This may also influence their interaction with effector cells and may enhance their suppressive function.

Apart from the IFN-γ that is produced at the inflammation site, we also observed Tregs and their intrinsic IFN-γ gene in both samples (CRC- and P-derived Tregs). This observation is in line with increasing evidence indicating that intrinsic IFN-γ Tregs play a major role in both inductions of Tregs as well as immunosuppression (38, 39). Studies have shown that IFN-γ is produced from stable Foxp3^+^ Tregs and has an essential immune-regulatory function in preventing experimental graft-versus-host disease (38). IFN-γ Tregs develop rapidly during inflammation and represent the first line of Tregs that suppress initial immune responses (39). This supports our findings that Tregs-intrinsic IFN-γ production was essential for their immune suppressive function.

We took our analysis further in order to distinguish the genes that are only expressed in CRC- or P-derived Tregs using N-derived Tregs as the baseline. We found that CCR2 was highly expressed on CRC- but not in P-derived Tregs. CCR2 binds the ligand (CCL2) to exert its activity. Tumor environment has been reported to express a high level of CCL2 (40, 41). We postulate that CCR2 is important for Tregs migration to the cancer site. This may activate CCL2/CCR2 signaling pathway and is important in promoting tumor growth.

We also observed an overexpression of SOCS3 in Tregs derived from CRC. Overexpression of SOCS3 is correlated with decreased proliferation and suppressive function (42). SOCS3 binds to the Janus kinases directly inhibiting their kinase activity (43). It could be that upregulation of the SOCS3 gene was in response to cytokine stimulation in the CRC environment. It may be that the SOCS proteins produced by Tregs inhibit cytokine-induced signaling pathways in T effector cells or other immune cells. This is supported by other studies that show a strong correlation of SOCS3 in T cells that inhibit Th1 but promote Th2 development (44, 45).

We detected high CCR7 expression in Tregs derived from P compared to CRC population. CCR7 is used by Tregs for homing into lymph nodes (LNs), where they expand upon antigen stimulation and inhibit the generation of antigen-specific T cells and suppress effector cell responses (46). It is possible that LN homing is part of their homing mechanism used by all Tregs and its absence may hamper Tregs’ localization into functional LN microenvironments.

We also observed high expression of CCL1 in P-derived Tregs. CCL1 is highly differentially regulated in Tregs in the presence of IL-6 (47). Neutralization of this chemokine ligand decreased Treg numbers and inhibited Tregs’ conversion and suppressive function (47). Increased expression of CCL1 Tregs could be one of the factors that interrupt APCs/T cells interaction. This supports the import function of CCL1 in Treg suppression.

Distinct TCR repertoires are displayed by Treg populations. In our study, we observed the downregulation of TCR in both CRC- and P-derived Tregs. This is in agreement with other studies that described Foxp3 to potently repress some TCR-induced genes, as well as some genes that are involved in the TCR signaling pathway (48, 49). Indeed, the downregulation of TCR in Tregs does not affect their expansion, activity, and function. TCR was largely dispensable for Foxp3 expression, lineage stability, and for high expression of many Treg signature genes (49). This support our data that TCR may not be needed for Tregs’ suppression function in CRC and P patients.

In summary, the present study reports a distinct transcriptomic profile of CD4^+^CD25^+^CD127^low^FoxP3^+^ Tregs derived from human CRC, P, and N. We suggest that the molecular signatures identified here may be important for Tregs function in homeostasis and immune suppression. We showed that the transcripts for cytokines, chemokines, and their receptors were highly expressed in CRC- and P-derived Tregs. This suggests their critical role for trafficking and regulating Tregs in CRC and P patients. The study may provide a better understanding of the immunomodulatory nature of Tregs and could, therefore, open up new avenues for immunotherapy.

## Ethics Statement

Ethics approval was obtained from the UKM Research Ethics Committee (Reference number UKM 1.5.3.5/244/FRGS/2/2013/SKK01/UKM/03/3). Participation in this study is voluntary. If the participants agree to take part, then they will be asked to sign the “Informed Consent Form.” They will be given a copy of the form and the information sheet on the study. The participant’s treatment at the hospital is not affected if they decide not to participate in this study. Should they decide to participate, they can still withdraw from the study without penalty. The data will not be used and will be discarded. The researcher may also remove the participants from the study for a variety of reasons. In this event, the participants will not be penalized or lose their rights as a patient.

## Author Contributions

NJ and RJ: conception or design of the work; or NJ, IS: the acquisition, NJ: analysis, or NJ and KT: interpretation of data for the work; and NJ, KT, and RJ: drafted the work or revised it critically for important intellectual content; and NJ, KT, IS, and RJ: final approval of the version to be published, and agreement to be accountable for all aspects of the work in ensuring that questions related to the accuracy or integrity of any part of the work are appropriately investigated and resolved.

## Conflict of Interest Statement

The authors declare that the research was conducted in the absence of any commercial or financial relationships that could be construed as a potential conflict of interest.

## References

[1] Sakaguchi S Hori S Fukui Y Sasazuki T Sakaguchi N Takahashi T. Thymic generation and selection of CD25+CD4+ regulatory T cells: implications of their broad repertoire and high self-reactivity for the maintenance of immunological self-tolerance. Novartis Found Symp (2003) 252:6–16; discussion 23, 106–14. 10.1002/0470871628.ch2 14609209

[2] Chaput N Louafi S Bardier A Charlotte F Vaillant JC Ménégaux F Identification of CD8+CD25+Foxp3+ suppressive T cells in colorectal cancer tissue. Gut (2009) 58(4):520–9. 10.1136/gut.2008.158824 19022917

[3] Endharti AT Okuno Y Shi Z Misawa N Toyokuni S Ito M CD8+CD122+ regulatory T cells (Tregs) and CD4+ Tregs cooperatively prevent and cure CD4+ cell-induced colitis. J Immunol (2011) 186(1):41–52. 10.4049/jimmunol.1000800 21098236

[4] Zhou J Ding T Pan W Zhu LY Li L Zheng L. Increased intratumoral regulatory T cells are related to intratumoral macrophages and poor prognosis in hepatocellular carcinoma patients. Int J Cancer (2009) 125(7):1640–8. 10.1002/ijc.24556 19569243

[5] Chen KJ Lin SZ Zhou L Xie HY Zhou WH Taki-Eldin A Selective recruitment of regulatory T cell through CCR6-CCL20 in hepatocellular carcinoma fosters tumor progression and predicts poor prognosis. PLoS One (2011) 6(9):e24671. 10.1371/journal.pone.0024671 21935436 PMC3173477

[6] Xu L Xu W Qiu S Xiong S. Enrichment of CCR6+Foxp3+ regulatory T cells in the tumor mass correlates with impaired CD8+ T cell function and poor prognosis of breast cancer. Clin Immunol (2010) 135(3):466–75. 10.1016/j.clim.2010.01.014 20181533

[7] Liotta F Gacci M Frosali F Querci V Vittori G Lapini A Frequency of regulatory T cells in peripheral blood and in tumour-infiltrating lymphocytes correlates with poor prognosis in renal cell carcinoma. BJU Int (2011) 107(9):1500–6. 10.1111/j.1464-410X.2010.09555.x 20735382

[8] Shenghui Z Yixiang H Jianbo W Kang Y Laixi B Yan Z Elevated frequencies of CD4^+^ CD25^+^ CD127lo regulatory T cells is associated to poor prognosis in patients with acute myeloid leukemia. Int J Cancer (2011) 129(6):1373–81. 10.1002/ijc.25791 21105040

[9] Salama P Phillips M Grieu F Morris M Zeps N Joseph D Tumor-infiltrating FOXP3+ T regulatory cells show strong prognostic significance in colorectal cancer. J Clin Oncol (2009) 27(2):186–92. 10.1200/JCO.2008.18.7229 19064967

[10] Haas M Dimmler A Hohenberger W Grabenbauer GG Niedobitek G Distel LV Stromal regulatory T-cells are associated with a favorable prognosis in gastric cancer of the cardia. BMC Gastroenterol (2009) 9:65 10.1186/1471-230X-9-65 19732435 PMC2749861

[11] Pretscher D Distel LV Grabenbauer GG Wittlinger M Buettner M Niedobitek G Distribution of immune cells in head and neck cancer: CD8+ T-cells and CD20+ B-cells in metastatic lymph nodes are associated with favorable outcome in patients with oro- and hypopharyngeal carcinoma. BMC Cancer (2009) 9:292 10.1186/1471-2407-9-292 19698134 PMC2739224

[12] Abbas AK Benoist C Bluestone JA Campbell DJ Ghosh S Hori S Regulatory T cells: recommendations to simplify the nomenclature. Nat Immunol (2013) 14(4):307–8. 10.1038/ni.2554 23507634

[13] Yu N Li X Song W Li D Yu D Zeng X CD4(+)CD25 (+)CD127 (low/-) T cells: a more specific Treg population in human peripheral blood. Inflammation (2012) 35(6):1773–80. 10.1007/s10753-012-9496-8 22752562

[14] Lim KP Chun NAL Ismail SM Abraham MT Yusoff MN Zain RB CD4^+^CD25^hi^CD127^low^ Regulatory T Cells Are Increased in Oral Squamous Cell Carcinoma Patients. PLoS One (2014) 9(8):e103975 10.1371/journal.pone.0103975 25153698 PMC4143252

[15] Shen LS Wang J Shen DF Yuan XL Dong P Li MX CD4(+)CD25(+)CD127(low/-) regulatory T cells express Foxp3 and suppress effector T cell proliferation and contribute to gastric cancers progression. Clin Immunol (2009) 131(1):109–18. 10.1016/j.clim.2008.11.010 19153062

[16] Stewart CA Metheny H Iida N Smith L Hanson M Steinhagen F Interferon-dependent IL-10 production by Tregs limits tumor Th17 inflammation. J Clin Invest (2013) 123(11):4859–74. 10.1172/JCI65180 24216477 PMC3809773

[17] Laidlaw BJ Cui W Amezquita RA Gray SM Guan T Lu Y Production of IL-10 by CD4(+) regulatory T cells during the resolution of infection promotes the maturation of memory CD8(+) T cells. Nat Immunol (2015) 16(8):871–9. 10.1038/ni.3224 26147684 PMC4713030

[18] Tran DQ. TGF-β: the sword, the wand, and the shield of FOXP3(+) regulatory T cells. J Mol Cell Biol (2012) 4(1):29–37. 10.1093/jmcb/mjr033 22158907

[19] Stewart BW Wild CP World Cancer Report 2014. Lyon, France: International Agency for Research on Cancer (2014). 39432694

[20] Omar ZA Ibrahim Tamin NS National Cancer Registry Report, Malaysia Cancer Statistics-Data and Figure 2007. Malaysia: Ministry of Health (2011).

[21] Hanahan D Weinberg RA Hallmarks of cancer: the next generation. Cell (2011) 144(5):646–74. 10.1016/j.cell.2011.02.013 21376230

[22] Hus I Bojarska-Junak A Chocholska S Tomczak W Woś J Dmoszyńska A Th17/IL-17A might play a protective role in chronic lymphocytic leukemia immunity. PLoS One (2013) 8(11):e78091 10.1371/journal.pone.0078091 24223764 PMC3815235

[23] de Bruin EC van de Velde CJ van Krieken JH Marijnen CA Medema JP. Epithelial human leukocyte antigen-DR expression predicts reduced recurrence rates and prolonged survival in rectal cancer patients. Clin Cancer Res (2008) 14(4):1073–9. 10.1158/1078-0432.CCR-07-1597 18281539

[24] Chaudhuri S Cariappa A Tang M Bell D Haber DA Isselbacher KJ Genetic susceptibility to breast cancer: HLA DQB*03032 and HLA DRB1*11 may represent protective alleles. Proc Natl Acad Sci U S A (2000) 97(21):11451–4. 10.1073/pnas.97.21.11451 11027344 PMC17220

[25] Hazewinkel Y de Wijkerslooth TR Stoop EM Bossuyt PM Biermann K van de Vijver MJ Prevalence of serrated polyps and association with synchronous advanced neoplasia in screening colonoscopy. Endoscopy (2014) 46(3):219–24. 10.1055/s-0033-1358800 24254386

[26] Ng SC Ching JY Chan VC Wong MC Tang R Wong S Association between serrated polyps and the risk of synchronous advanced colorectal neoplasia in average-risk individuals. Aliment Pharmacol Ther (2015) 41(1):108–15. 10.1111/apt.13003 25339583

[27] Urman J Gomez M Basterra M Mercado MD Montes M Gómez Dorronsoro M Serrated polyps and their association with synchronous advanced colorectal neoplasia. Gastroenterol Hepatol (2016) 39(9):574–83. 10.1016/j.gastrohep.2015.12.010 26973340

[28] Li D Jin C McCulloch C Kakar S Berger BM Imperiale TF Association of large serrated polyps with synchronous advanced colorectal neoplasia. Am J Gastroenterol (2009) 104(3):695–702. 10.1038/ajg.2008.166 19223889

[29] Huang CS Farraye FA Yang S O’Brien MJ. The clinical significance of serrated polyps. Am J Gastroenterol (2011) 106(2):229–40; quiz 41. 10.1038/ajg.2010.429 21045813

[30] Rex DK Ahnen DJ Baron JA Batts KP Burke CA Burt RW Serrated lesions of the colorectum: review and recommendations from an expert panel. Am J Gastroenterol (2012) 107(9):1315–29; quiz 4, 30. 10.1038/ajg.2012.161 22710576 PMC3629844

[31] Shen LS Wang J Shen DF Yuan XL Dong P Li MX CD4+CD25+CD127low/- regulatory T cells express Foxp3 and suppress effector T cell proliferation and contribute to gastric cancers progression. Clin Immunol (2009) 131(1):109–18. 10.1016/j.clim.2008.11.010 19153062

[32] Betts G Jones E Junaid S El-Shanawany T Scurr M Mizen P Suppression of tumour-specific CD4^+^ T cells by regulatory T cells is associated with progression of human colorectal cancer. Gut (2012) 61(8):1163–71. 10.1136/gutjnl-2011-300970 22207629 PMC3388728

[33] Molinaro R Pecli C Guilherme RF Alves-Filho JC Cunha FQ Canetti C CCR4 controls the suppressive effects of regulatory T cells on early and late events during severe sepsis. PLoS One (2015) 10(7):e0133227. 10.1371/journal.pone.0133227 26197455 PMC4511514

[34] Gobert M Treilleux I Bendriss-Vermare N Bachelot T Goddard-Leon S Arfi V Regulatory T cells recruited through CCL22/CCR4 are selectively activated in lymphoid infiltrates surrounding primary breast tumors and lead to an adverse clinical outcome. Cancer Res (2009) 69(5):2000–9. 10.1158/0008-5472.CAN-08-2360 19244125

[35] Sugiyama D Nishikawa H Maeda Y Nishioka M Tanemura A Katayama I Anti-CCR4 mAb selectively depletes effector-type FoxP3+CD4+ regulatory T cells, evoking antitumor immune responses in humans. Proc Natl Acad Sci U S A (2013) 110(44):17945–50. 10.1073/pnas.1316796110 24127572 PMC3816454

[36] Ishida T Ishii T Inagaki A Yano H Komatsu H Iida S Specific recruitment of CC chemokine receptor 4-positive regulatory T cells in Hodgkin lymphoma fosters immune privilege. Cancer Res (2006) 66(11):5716–22. 10.1158/0008-5472.CAN-06-0261 16740709

[37] Lunardi S Jamieson NB Lim SY Griffiths KL Carvalho-Gaspar M Al-Assar O IP-10/CXCL10 induction in human pancreatic cancer stroma influences lymphocytes recruitment and correlates with poor survival. Oncotarget (2014) 5(22):11064–80. 10.18632/oncotarget.2519 25415223 PMC4294325

[38] Koenecke C Lee CW Thamm K Föhse L Schafferus M Mittrücker HW IFN-γ production by allogeneic Foxp3+ regulatory T cells is essential for preventing experimental graft-versus-host disease. J Immunol (2012) 189(6):2890–6. 10.4049/jimmunol.1200413 22869903

[39] Daniel V Wang H Sadeghi M Opelz G. Interferon-gamma producing regulatory T cells as a diagnostic and therapeutic tool in organ transplantation. Int Rev Immunol (2014) 33(3):195–211. 10.3109/08830185.2013.845181 24266365

[40] Chun E Lavoie S Michaud M Gallini CA Kim J Soucy G CCL2 promotes colorectal carcinogenesis by enhancing polymorphonuclear myeloid-derived suppressor cell population and function. Cell Rep (2015) 12(2):244–57. 10.1016/j.celrep.2015.06.024 26146082 PMC4620029

[41] Kitamura T Qian BZ Soong D Cassetta L Noy R Sugano G CCL2-induced chemokine cascade promotes breast cancer metastasis by enhancing retention of metastasis-associated macrophages. J Exp Med (2015) 212(7):1043–59. 10.1084/jem.20141836 26056232 PMC4493415

[42] Pillemer BB Xu H Oriss TB Qi Z Ray A. Deficient SOCS3 expression in CD4+CD25+FoxP3+ regulatory T cells and SOCS3-mediated suppression of Treg function. Eur J Immunol (2007) 37(8):2082–9. 10.1002/eji.200737193 17621372

[43] Sasaki A Yasukawa H Suzuki A Kamizono S Syoda T Kinjyo I Cytokine-inducible SH2 protein-3 (CIS3/SOCS3) inhibits Janus tyrosine kinase by binding through the N-terminal kinase inhibitory region as well as SH2 domain. Genes Cells (1999) 4(6):339–51. 10.1046/j.1365-2443.1999.00263.x 10421843

[44] Seki Y Inoue H Nagata N Hayashi K Fukuyama S Matsumoto K SOCS-3 regulates onset and maintenance of T(H)2-mediated allergic responses. Nat Med (2003) 9(8):1047–54. 10.1038/nm896 12847520

[45] White CA Nicola NA. SOCS3: an essential physiological inhibitor of signaling by interleukin-6 and G-CSF family cytokines. JAKSTAT (2013) 2(4):e25045. 10.4161/jkst.25045 24416642 PMC3876435

[46] Schneider MA Meingassner JG Lipp M Moore HD Rot A. CCR7 is required for the in vivo function of CD4+ CD25+ regulatory T cells. J Exp Med (2007) 204(4):735–45. 10.1084/jem.20061405 17371928 PMC2118557

[47] Hoelzinger DB Smith SE Mirza N Dominguez AL Manrique SZ Lustgarten J. Blockade of CCL1 inhibits T regulatory cell suppressive function enhancing tumor immunity without affecting T effector responses. J Immunol (2010) 184(12):6833–42. 10.4049/jimmunol.0904084 20483762

[48] Ouyang W Liao W Luo CT Yin N Huse M Kim MV Novel Foxo1-dependent transcriptional programs control T(reg) cell function. Nature (2012) 491(7425):554–9. 10.1038/nature11581 23135404 PMC3771531

[49] Levine AG Arvey A Jin W Rudensky AY. Continuous requirement for the TCR in regulatory T cell function. Nat Immunol (2014) 15(11):1070–8. 10.1038/ni.3004 25263123 PMC4205268

